# GenomeCAT: a versatile tool for the analysis and integrative visualization of DNA copy number variants

**DOI:** 10.1186/s12859-016-1430-x

**Published:** 2017-01-06

**Authors:** Katrin Tebel, Vivien Boldt, Anne Steininger, Matthias Port, Grit Ebert, Reinhard Ullmann

**Affiliations:** 1 Max Planck Institute for Molecular Genetics, 14195 Berlin, Germany; 2 Department of Biology, Chemistry and Pharmacy, Free University Berlin, 14195 Berlin, Germany; 3 Institut für Radiobiologie der Bundeswehr in Verb. mit der Universität Ulm, 80937 Munich, Germany

**Keywords:** DNA copy number variants, Integrative visualization, Microarray, NGS

## Abstract

**Background:**

The analysis of DNA copy number variants (CNV) has increasing impact in the field of genetic diagnostics and research. However, the interpretation of CNV data derived from high resolution array CGH or NGS platforms is complicated by the considerable variability of the human genome. Therefore, tools for multidimensional data analysis and comparison of patient cohorts are needed to assist in the discrimination of clinically relevant CNVs from others.

**Results:**

We developed GenomeCAT, a standalone Java application for the analysis and integrative visualization of CNVs. GenomeCAT is composed of three modules dedicated to the inspection of single cases, comparative analysis of multidimensional data and group comparisons aiming at the identification of recurrent aberrations in patients sharing the same phenotype, respectively. Its flexible import options ease the comparative analysis of own results derived from microarray or NGS platforms with data from literature or public depositories. Multidimensional data obtained from different experiment types can be merged into a common data matrix to enable common visualization and analysis. All results are stored in the integrated MySQL database, but can also be exported as tab delimited files for further statistical calculations in external programs.

**Conclusions:**

GenomeCAT offers a broad spectrum of visualization and analysis tools that assist in the evaluation of CNVs in the context of other experiment data and annotations. The use of GenomeCAT does not require any specialized computer skills. The various R packages implemented for data analysis are fully integrated into GenomeCATs graphical user interface and the installation process is supported by a wizard. The flexibility in terms of data import and export in combination with the ability to create a common data matrix makes the program also well suited as an interface between genomic data from heterogeneous sources and external software tools. Due to the modular architecture the functionality of GenomeCAT can be easily extended by further R packages or customized plug-ins to meet future requirements.

**Electronic supplementary material:**

The online version of this article (doi:10.1186/s12859-016-1430-x) contains supplementary material, which is available to authorized users.

## Background

DNA copy number variants represent the greatest source of genetic variability in humans [1] and are the underlying cause of many human diseases. Array CGH is recognized as a first-tier test for DNA copy number variants (CNV) [2] and accordingly, many laboratories have already established their pipelines for pre-processing of array CGH data and CNV calling. In many cases these pipelines are based on software packages provided by the companies selling DNA microarrays or scanners such as BlueFuse [3], CytoSure [4] or CytoGenomics [5]. Yet, the scope of these tools is focused on the identification of CNVs and their evaluation in the context of gene content and frequency of a given variant in the healthy population. Comparative analysis, which integrates data obtained from multiple patients, or other experiment types are hardly supported, in particular when they are based on different array platforms or NGS technology.

Such kind of meta-analysis needs the implementation of additional commercial or free software. Each of the currently existing software solutions have their particular strength and focus. Some are particularly useful for the identification of genomic regions significantly associated with a given phenotype [4, 6–12] or have implemented algorithms specifically designed to detect and query copy number changes in SNP data sets [8, 13]. Others provide a gene centered view on copy number aberrations [14, 15] or examine CNVs in a clinical context [16]. Only a few free software packages offer a comprehensive spectrum of visualization and analysis tools for multidimensional array data operable via a graphical user interface [12–17]. What these tools have in common is that they have been designed with the intention to analyze microarray data. NGS data are usually displayed in alternative data browsers such as the Integrative Genome Viewer - IGV [18], or the Integrated Genome Browser – IGB [19]. These browsers also support visualization of array data when present in the appropriate format. However, as in the case of the IGV, analysis of array data that goes beyond visualization requires the export to the GenePattern software [20], where several web-based features for DNA copy number analysis are provided.

In light of the increasing relevance of multi-dimensional data analysis several commercial softwares have been brought to market, including Partek [21], GenomicWorkbench [22], Genedata Expressionist for Genomic Profiling [23], Array Studio [24], GenomeStudio [25], CGH Fusion [26], Nexus Expression [27], CLC Workbench [28] and Subio [29]. Yet, these programs are neither open source nor free in most instances. Thus, considerable licensing fees have to be paid and advancement of this software is solely dependent on the company.

Proceeding on the experiences with our previous analysis software CGHPRO [30], we aimed to create a versatile tool that facilitates the meta-analysis of array CGH results and corresponding data from other experiment types and platforms. We designed GenomeCAT under the premise that it is easy to install and use, and offers a broad spectrum of flexible visualization and analysis options without the need of specialized computer skills or the obligation to upload sensible patient data to web servers.

## Implementation

### Software architecture

**Fig. 1:**
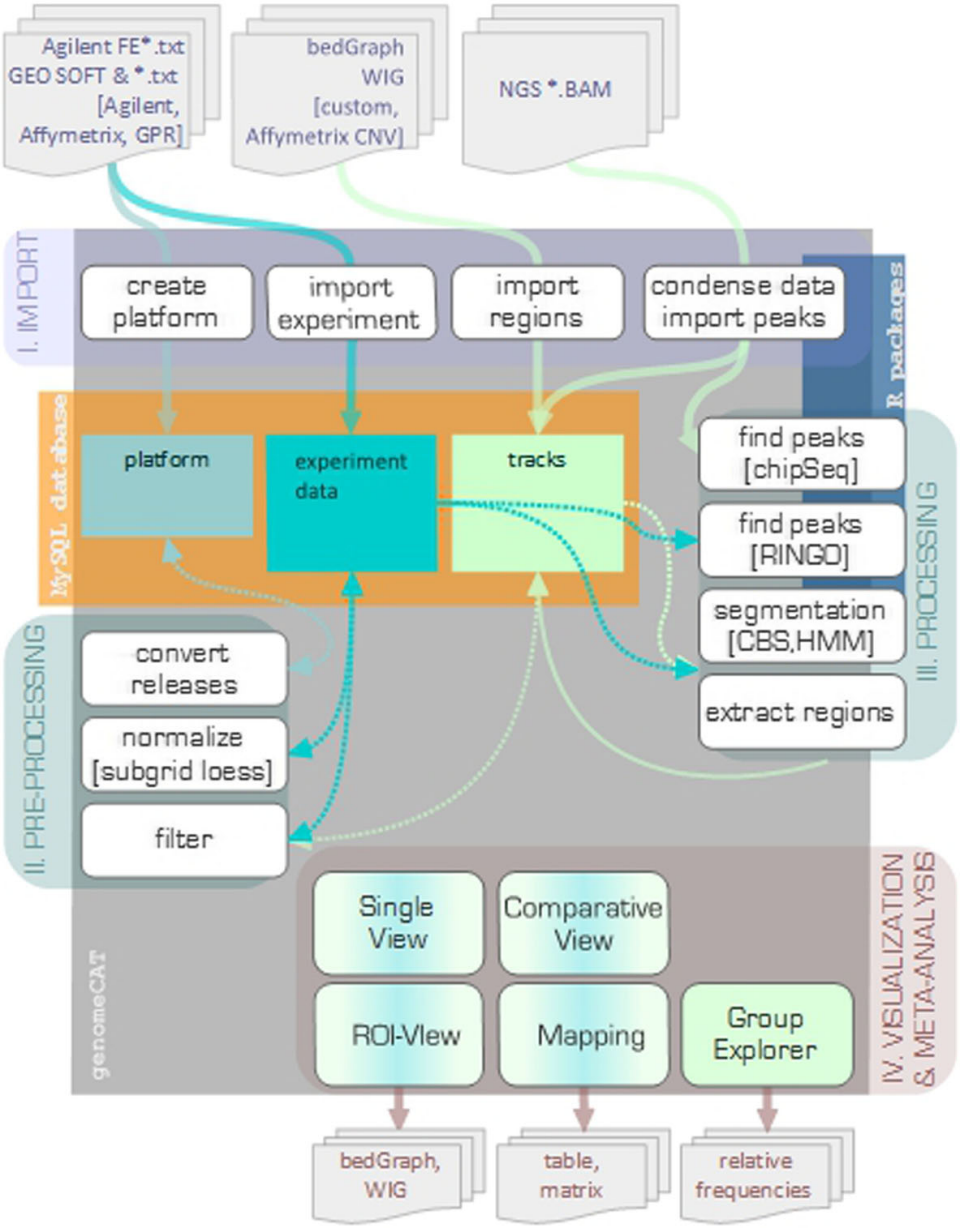
Schematic presentation of the workflow in GenomeCAT

GenomeCAT is a desktop application developed in Java using the NetBeans Platform. It is an open source software and is provided as a free download. The program has a modular structure, which supports the program-aided updating and the implementation of new plug-ins. At the center of the program is a MySQL database, designed to maintain experiment data, metadata and annotation tracks. The current version refers to the human genome only, but the database is designed to be adaptable to any other genome when necessary (Fig. 1). An installation wizard guides through the installation process that comprises the set-up of the desktop application, the MySQL database and an R environment.

The execution of R packages is embedded in the desktop application. Users can enter and modify method-specific parameters via a dialog box. The R packages themselves run as a background process, the progress of which is reported on the screen (Additional file 1). Results produced by the R session are automatically stored into GenomeCAT. The design of this interface eases the future addition of R packages in order to further increase the functionality of GenomeCAT.

### Integration of heterogeneous data

Multidimensional data are frequently produced by means of different experiment platforms. This implies that comparative analysis has to be preceded by the creation of a common data matrix. GenomeCAT is capable to address this issue in three ways: data binning based on annotation attributes (for example genes), genomic bins of variable size or user defined intervals. The resulting data matrix can not only be accessed within GenomeCAT, but can also be exported for further analysis in external programs (e.g., to visualize data as interactive heatmaps in Gitools [31] or as network attributes in Cytoscape [32]).

### Performance issues

The search for features overlapping with a given genomic interval is a recurrent procedure in multidimensional data analysis. This applies also to the mapping to annotation attributes or genomic intervals as described above. In order to accelerate these queries in GenomeCAT we took advantage of the Spatial Index, an extension of the MySQL database. There, genomic locations or intervals are stored as geometric objects, which are indexed via R-Trees [33]. Based on this indexing scheme we employ the MBRIntersect function [34, 35] for queries and filters, which speeds up the processing time by a factor of 4 on average compared to the use of a composite index. In contrast to other software packages [36] in GenomeCAT the number of cases that can be simultaneously analyzed is not confined. It is only limited by the heapsize of the Java application. Loading of data and computational intensive calculations are parallelized in order to optimally exploit the potential of the multi-core architecture of modern CPUs.

## Results and Discussion

### Data import

GenomeCAT supports different ways of data import. While users can choose the traditional way – to import an array platform and add sample data afterwards - the preferred route may be the direct import of data formatted in BED style (chromosome start stop score). This format is simple, platform independent and enables more flexible entry points into the analysis with GenomeCAT. Thus users can stick to their well-established pipeline for primary data analysis and start the analysis in our software with a set of already predefined CNVs. Moreover, the format is ideal for comparisons of own array CGH data with results from other experiment types or, for example, CNVs that have been reported in literature as a list of genomic intervals. For maximal flexibility, our software also offers the option to interactively compile the necessary data from more comprehensive tables.

In addition, GenomeCAT has import routines for experiment data available in GEOs SOFT file format and can also process BAM files for the import of NGS data. All data are stored in a MySQL database together with metadata such as phenotype information. Data are organized in a hierarchical structure that is searchable and can be filtered by various criteria.

### Module 1: single view

**Fig. 2:**
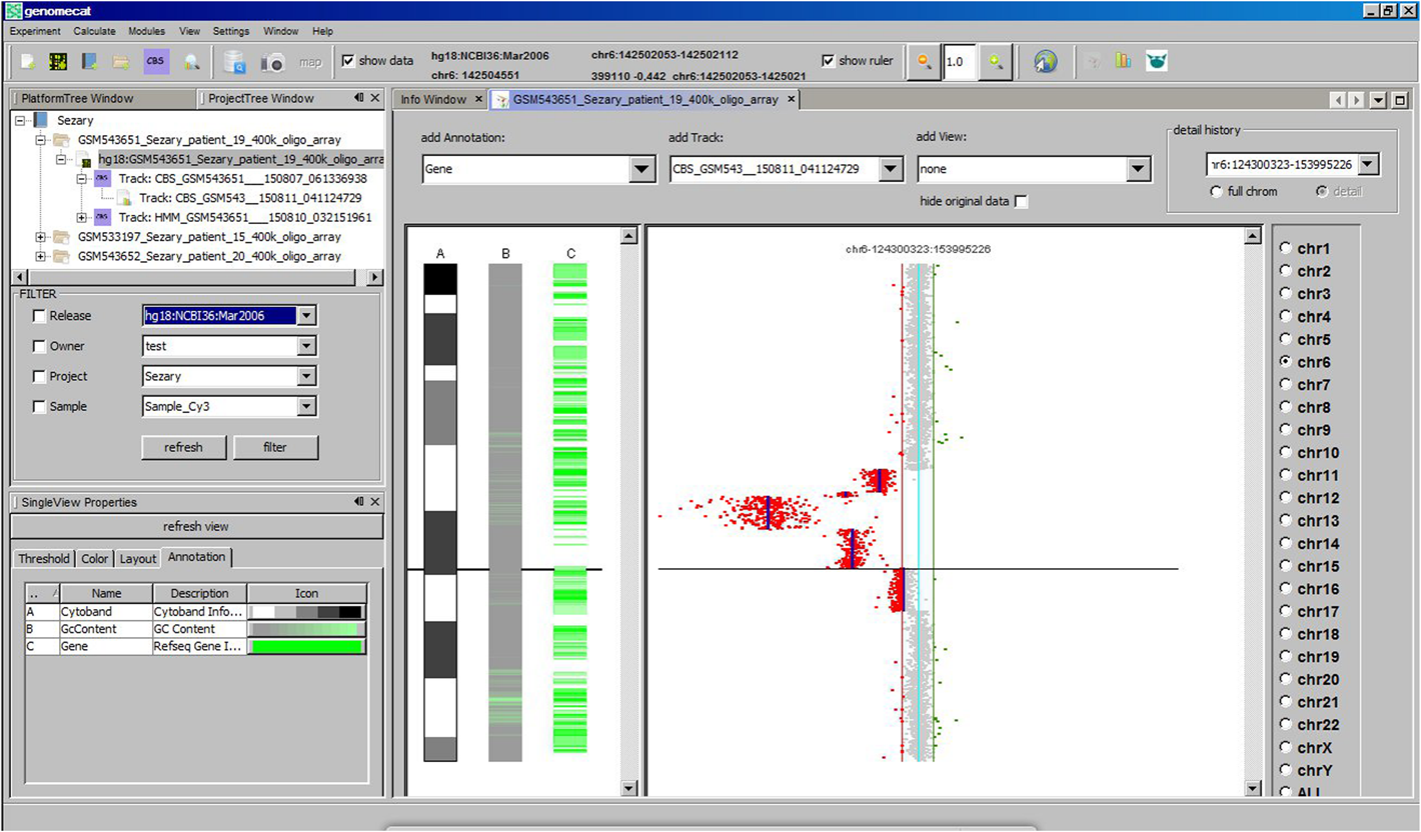
Graphical User Interphase of GenomeCAT in the Single View mode. Array CGH results for part of chromosome 6 in a patient with cutaneous T-cell lymphoma are depicted as familiar ratio plot together with a track highlighting the aberrant segments as detected by CBS analysis (black bars). Additionally, oligos with ratios beyond custom-defined thresholds are coloured in red and green, respectively. Note that chromosomal breakpoints correlate with transition from gene rich to gene poor regions as visualized by annotation track C right to the chromosome ideogram

Single case analysis can be accomplished with *SingleView*, the first of the three modules that make up GenomeCAT. In this module users can display array CGH profiles as familiar ratio plots along the chromosome ideograms. Optionally, annotation tracks such as GC content, CNVs from the Database of Genomic Variants [37] and segmental duplications [38] can be depicted beside (Fig. 2). The layout can be customized and the plots are interactive. For example, genomic coordinates are provided as mouseover event and regions can be zoomed-in or directly viewed in the UCSC Genome Browser [39]. CNV calling can be performed by means of customizable fixed and dynamic threshold settings and in combination with CBS [40] or HMM [10]. For each genomic region extracted in this way, GenomeCAT calculates a quality score by dividing the averaged ratio value within each extracted region by the median average deviation. If CBS has been employed before, the median average deviation is calculated based on the deviation from the averaged ratio value within the segments as defined by CBS. Moreover, Ringo [41] is implemented to facilitate peak finding in data derived from chromatin immunoprecipitation (ChIP) experiments. All processed data are stored as separate tracks and each processing step is recorded together with its parameter settings. Thus it is easy to recapitulate the analysis procedure and to go back to the original data if necessary, or to compare results obtained with different parameter settings. All tracks generated in the course of data analysis can be exported as tab delimited file in BED or BedGraph format, which is suitable for direct visualization in the UCSC Genome Browser [39].

**Fig. 3:**
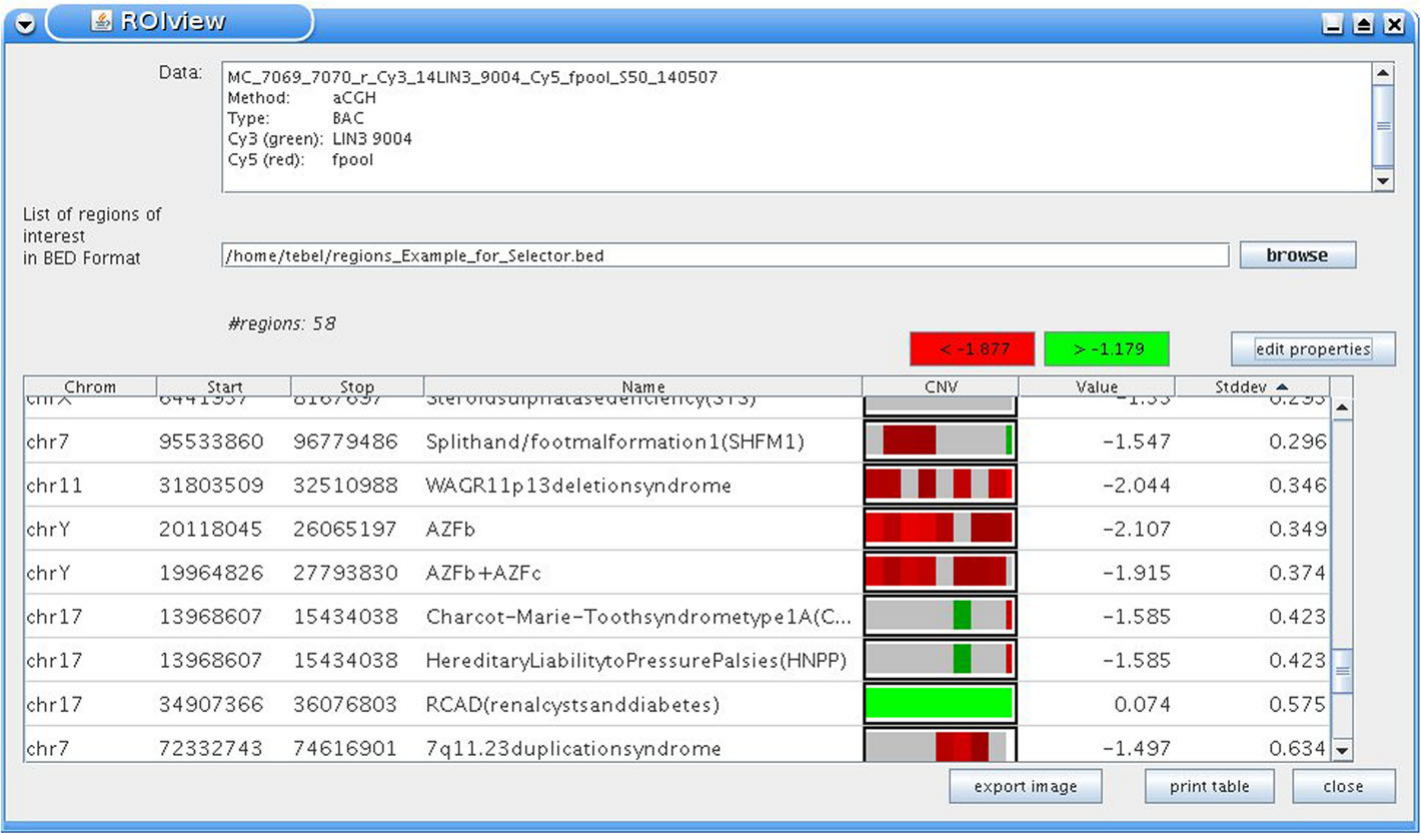
Region of Interest Viewer. For the purpose of demonstration a set of intervals recurrently altered in genomic disorders have been used to filter array CGH results of a breast carcinoma cell line. Each of these user-defined intervals is split into ten segments. These segments are visualized in the central column as heatmap with the ratio values defining colour and saturation (e.g., red: deletion, green: gain; grey: within thresholds). Average ratios and standard deviation of each interval are given in the columns to the right

A different type of single case visualization can be performed in the Region of Interest (ROI) viewer. Proceeding on a list of user defined genomic intervals this feature sorts these intervals according to their average experiment values and displays the value distribution of each defined interval in a heatmap with a resolution of 10 bins per interval. Applications of this feature can be the pre-screening of array CGH results based on a list of genomic intervals recurrently altered in genomic disorders (Fig. 3) or the identification of genes with highest scores in ChIP experiments to name a few.

### Module 2: comparative view

**Fig. 4:**
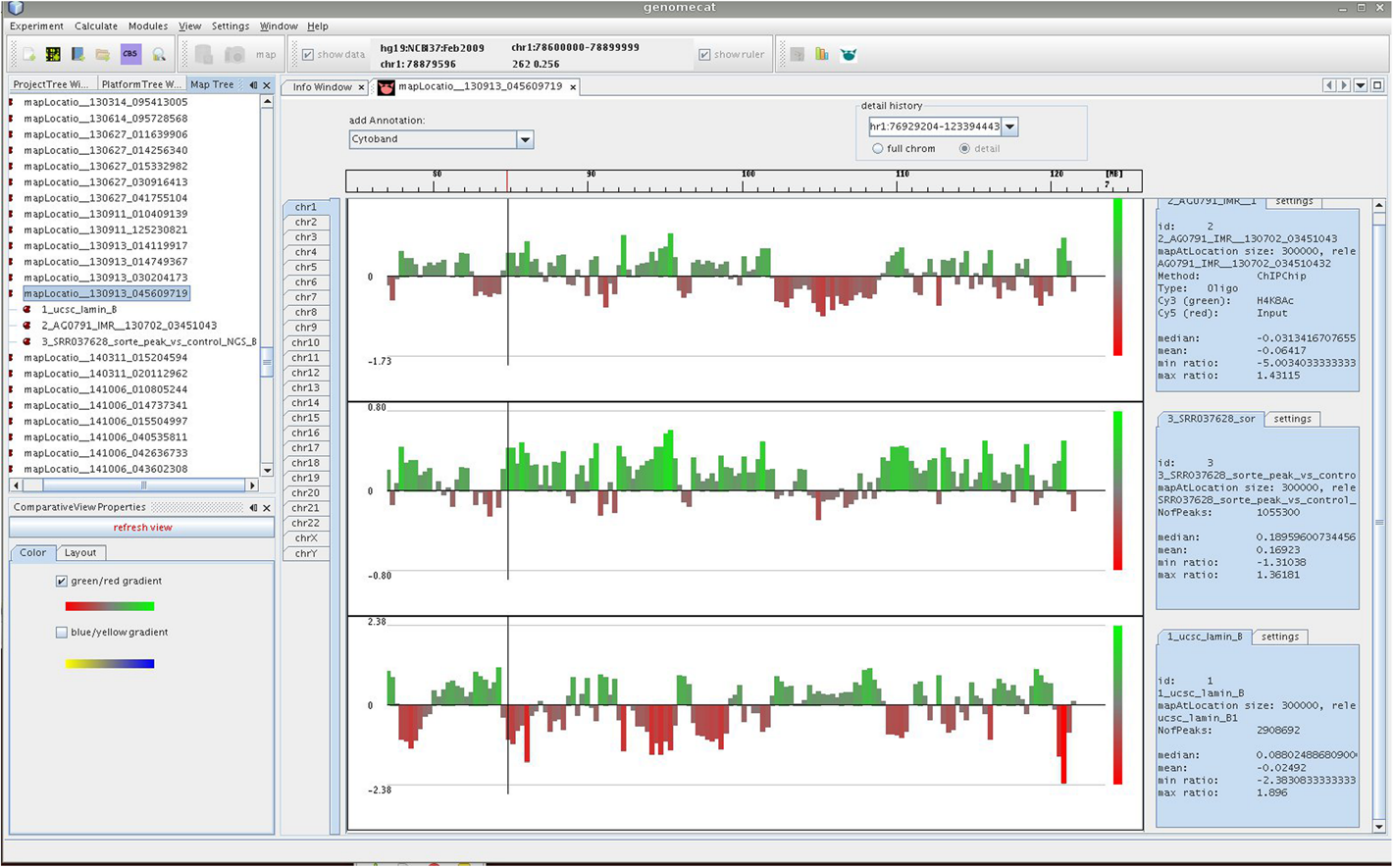
Comparative View. Data derived from different experiment types have been mapped to 100 kb intervals for simultanous visualization. Tracks can be resorted, zoomed-in, rescaled and intervals of interest can be checked in the UCSC genome browser. First track: microarray data H4K8ac; second track: NGS data H4K8ac; third track: public data set on lamin B1 [42].

The second module of GenomeCAT is dedicated to the simultaneous visualization of multiple tracks. These can be the results of the same sample processed with different parameters, data from the very same patient but different array or NGS-based experiment types or data from different patients. Plots produced by this module are interactive, including the options to re-sort, to zoom-in and re-scale, and to view the intervals of interest in the UCSC Genome Browser (Fig. 4).

One practical issue that complicates the integrative analysis of data derived from different experiment types is the fact, that they usually do not share a common coordinate system. Oligonucleotides of gene expression and CGH arrays hardly overlap and both platforms are not directly comparable to NGS data. This problem is addressed by our mapping feature, which allows the creation of a common data matrix for all experiments opened in this view either based on genomic bins of selectable size, genes or custom defined intervals. The resulting table is automatically stored in the internal database, but can also be exported as tab delimited file for down-stream analysis by statistical packages or visualization in other tools. For example, mapping on genes can be used to display array CGH ratios as attributes in a Cytoscape network.

### Module 3: group explorer

The considerable variability of the human genome in health and disease complicates the interpretation of CNVs or patterns of copy number alterations. Recurrence of aberrations within a group of patients with similar phenotype or differences between patient groups has proven a valuable criterion to filter for biological meaningful alterations. The third module of GenomeCAT has been designed to facilitate such group comparisons. All experiment results stored in the GenomeCAT database can be filtered and selected for simultaneous visualization based on metadata such as phenotype. Separate colors can be assigned to each group or even to individual cases. The latter option can be used to highlight particular cases to ease their identification in the overview later on. CNVs can be displayed as colored bars along the chromosomes with the option to control opacity and color saturation of a given CNV by its ratio or quality score. In this way it is possible to discriminate homozygous deletions from heterozygous ones and moderate gains of DNA from high copy amplifications, respectively. Also this graphical user interface is interactive. Clicking on individual CNVs in the plot highlights case details in the adjacent table and vice versa. Regions of interest can be zoomed-in or checked in the UCSC Genome Browser.

**Fig. 5:**
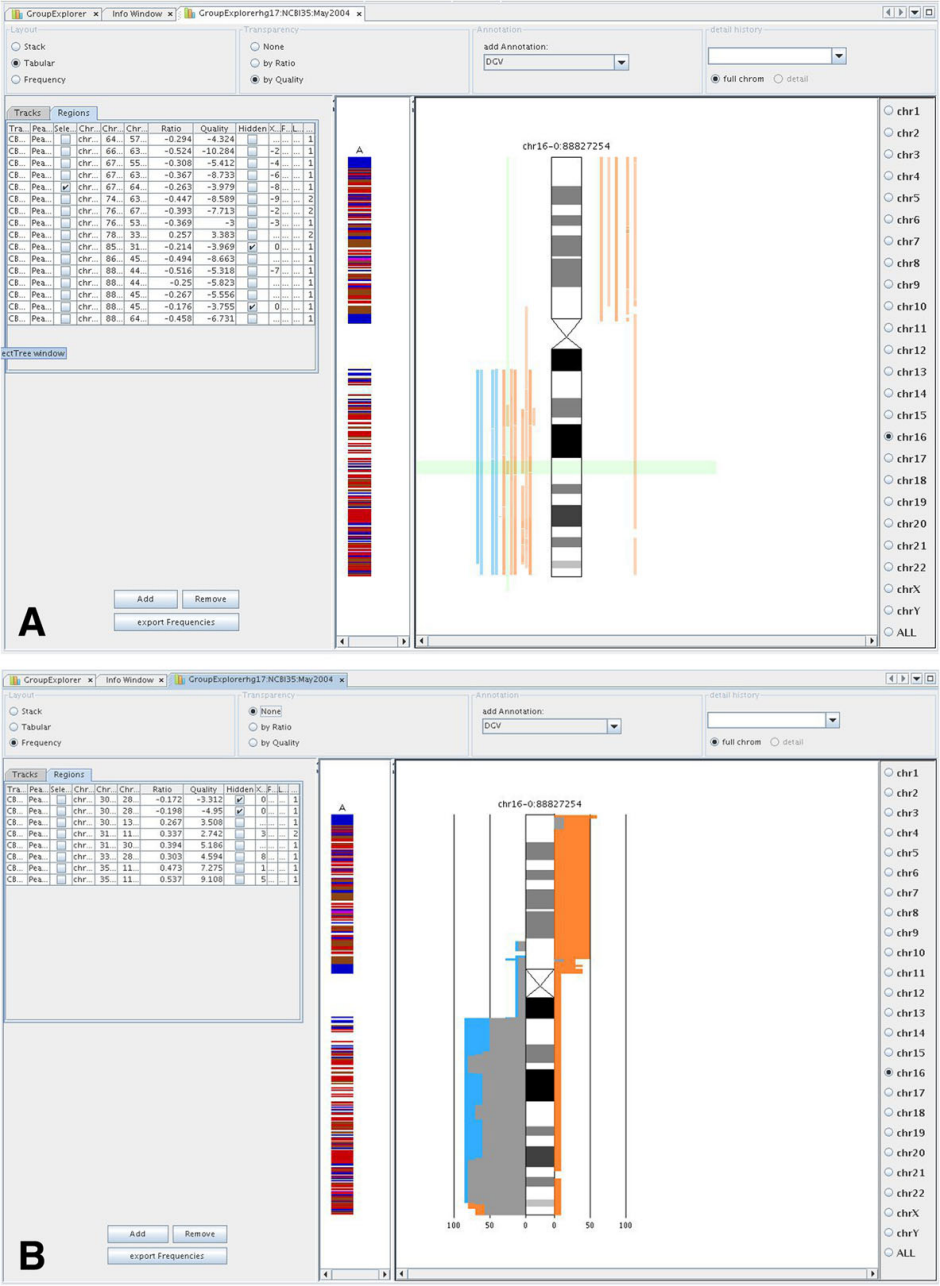
Absolute and relative frequencies of chromosomal aberrations depicted by means of the Group Explorer module. Comparison of chromosomal aberrations in two different types of breast cancer. **a** In the absolute view each aberration is given as vertical line to the left (deletions) and right (gains) of the chromosome ideogram in a tumor type specific colour. Ticking one box in the left table highlights the corresponding case in the absolute view and vice versa. **b** same cases in the relative view

While this mode is well suited to present the absolute numbers of CNVs and their genomic location, the relative frequency plot - also included in this module- can be employed to compare CNV frequencies independent of group size. As demonstrated in Fig. 5, this way of visualization facilitates the recognition of phenotype specific aberrations. However, the application of this tool is not restricted to CNV analysis, but it can also be used to depict the probability of epigenetic modifications in regions frequently affected by copy number changes in a specific tumor type and so forth. Relative frequencies as calculated by GenomeCAT can be exported as CSV files for further statistical analysis or visualization in external software packages.

## Conclusions

GenomeCAT provides comprehensive tools for the analysis of DNA copy number variants and facilitates the evaluation of their biological relevance in the context of genome annotations and results obtained from different experiment types. Its flexible import options ease the comparative analysis of own results with data from literature or public depositories. Moreover, GenomeCAT can act as an interface to other software tools since results generated in GenomeCAT can be exported in standard file formats.

## Availability and requirements

Project name: GenomeCAT

Project home page: http://genomecat.github.io/genomeCATSuite

Source code: https://github.com/genomeCAT/genomeCATSuite

Operating system: Linux and Windows 7

Programming language: Java, SQL, R

Other requirements: Java 1.8 or higher, MySQL database and R

License: GNU General Public License

Any restriction to use by non-academics: Contact authors

## Additional files

Additional file 1: Implementation of R Packages in GenomeCAT. Word document demonstrating how R packages are implemented in GenomeCAT. (DOCX 23 kb)

## Abbreviations

BAM: Binary alignment/map
BED: Browser extensible data
CBS: Circular binary segmentation
CGH: Comparative genomic hybridization
ChIP: Chromatin immunoprecipitation
CNV: Copy number variant
HMM: Hidden Markov Model
IGB: Integrated genome browser
IGV: Integrative genome viewer
MBR: Minimum bounding rectangle
ROI: Region of interest
