# Supplementary material for: GenomeCAT: a versatile tool for the analysis and integrative visualization of DNA copy number variants
Source: BMC Bioinformatics. 2017 Jan 6;18:19. doi: 10.1186/s12859-016-1430-x (PMC5217618; doi:10.1186/s12859-016-1430-x)
Supplement: Additional file 1: — Implementation of R Packages in GenomeCAT. Word document demonstrating how R packages are implemented in GenomeCAT. (DOCX 23 kb) [file 12859_2016_1430_MOESM1_ESM.docx]

**Implementation of R Packages in GenomeCAT**

**segment array cgh data with circular binary segmentation**

library(DNAcopy)
ddata<-read.table(file="/home/paula/GenomeCAT7//test_AG060__160708_033816279_hg18_Spots_cbs.dat", na.strings="\\N", header=TRUE, sep="\t")
data <- na.omit(ddata)
genomdat<-data$log2Ratio
chrom<-data$Chrom
maploc<-data$start
CNA.object<-CNA(genomdat,chrom,maploc,data.type="logratio")

#find breakpoints, segment data
result<-segment(CNA.object, verbose=3)
i <- sessionInfo()
write(paste("#Package:", i$otherPkgs$DNAcopy$Package), file="/home/paula/GenomeCAT7//test_AG060__160708_033816279_hg18_Spots_cbs.result")
write(paste("#Version:", i$otherPkgs$DNAcopy$Version), append=TRUE, file="/home/paula/GenomeCAT7//test_AG060__160708_033816279_hg18_Spots_cbs.result")
write(paste("#Reference:", gsub("\n", "", i$otherPkgs$DNAcopy$Reference)), append=TRUE, file="/home/paula/GenomeCAT7//test_AG060__160708_033816279_hg18_Spots_cbs.result")
write.table(result$output, append=TRUE, file="/home/paula/GenomeCAT7//test_AG060__160708_033816279_hg18_Spots_cbs.result", sep="\t",row.names=FALSE, col.names=FALSE)

**smooth array cgh data with Hidden Markov Model Algorithm**

library('aCGH', logical.return = TRUE)
ddata<-read.table(file="/home/paula/GenomeCAT7//test_AG060__160708_033816279_hg18_Spots_hmm.dat", na.strings="\\N", header=TRUE, sep=" ")
data <- na.omit(ddata)
log2.ratios<-data.frame(test=data$log2Ratio)
clone.info<-data.frame(Clone=data$Clone, Target=data$Target,Chrom=data$Chrom,kb=round(data$start/1000))
ex.acgh<-create.aCGH(log2.ratios, clone.info)

#computing HMM states
hmm(ex.acgh)<-find.hmm.states(ex.acgh, aic=TRUE, bic=FALSE)
y<-mergeLevels(ex.acgh$hmm$states.hmm[[1]][,8],ex.acgh$hmm$states.hmm[[1]][,6])
mad <- median(y$mnNow)
i <- sessionInfo()
write(paste("#Package:",i$otherPkgs$aCGH$Package), file="/home/paula/GenomeCAT7//test_AG060__160708_033816279_hg18_Spots_hmm.result")
write(paste("#Version:", i$otherPkgs$aCGH$Version), append=TRUE, file="/home/paula/GenomeCAT7//test_AG060__160708_033816279_hg18_Spots_hmm.result")
write(paste("#Reference:", gsub("\n", "", i$otherPkgs$aCGH$Reference)), append=TRUE, file="/home/paula/GenomeCAT7//test_AG060__160708_033816279_hg18_Spots_hmm.result")
write(paste("#MAD:", mad), append=TRUE, file="/home/paula/GenomeCAT7//test_AG060__160708_033816279_hg18_Spots_hmm.result")
out <- data.frame(data, ex.acgh$hmm$states.hmm[[1]])
out <- data.frame(out, y$vecMerged)
write.table(out, file="/home/paula/GenomeCAT7//test_AG060__160708_033816279_hg18_Spots_hmm.result", row.names=FALSE, col.names=FALSE, append=TRUE, sep=" ")

**find ChIP enriched Regions on array cgh data with RINGO**

#According to the tutorial we implemented normalizing “preprocess(..., method="nimblegen”,..)” and smoothing #“computeRunningMedians” of the data. RINGO's “findChersOnSmoothed” detects regions consisting of features #exceeding a certain threshold. If no user defined threshold is set we use the minimum of #“upperBoundNull(exprs(smoothX)” and “twoGaussiansNull(exprs(smoothX))”. If no probe distance is set by user #we calculate it based on the median distance of the features. In our tests we found feature density the most #critical point of this analysis.

library('Ringo')
RG3 <-read.maimages('/home/paula/GenomeCAT7//test_AG060__160708_033816279_hg18_Spots_RINGO.dat', columns=list(R="GSignal",G="RSignal") , source="generic", annotation=c("ProbeName","SystematicName", "GeneName"))
targets <- data.frame(FileName=I(c("test_AG060__160708_033816279_hg18_Spots_RINGO.R")),Cy3=I(c("sample")),Cy5=I(c("control")))
row.names(targets) <- c("test_AG0605_MCIp_Cy3_3h_DMSO_Cy5_0h_OB")
RG3$targets <- targets
X <- preprocess(RG3, method="nimblegen", idColumn="ProbeName")
pA <- extractProbeAnno(RG3, "agilent", genome="human", microarray="Agilent Tiling ")
 d <- signif(median(diff(pA["1.start"])), d=1)
dist <- d*3/2; d
nprobes <- 3;nprobes
smoothX <- computeRunningMedians(X, modColumn="Cy3", winHalfSize=d, min.probes=nprobes, probeAnno=pA)
y0G <- twoGaussiansNull(exprs(smoothX));y0G
y0 <- upperBoundNull(exprs(smoothX));y0
y0G <- min(y0, y0G);y0G
png("/home/paula/GenomeCAT7//test_AG060__160708_033816279_hg18_Spots_RINGO.png")
hist(exprs(smoothX), n=100, main=NA, xlab="Smoothed expression level [log2]")
 abline(v=y0G, col="blue")
legend(x="topright", lwd=1, col=c("blue"),legend=c("Threshold"))
dev.off()
chersX <- findChersOnSmoothed(smoothX, distCutOff=4*dist,minProbesInRow=nprobes, probeAnno=pA, threshold=y0G)
chersXD <- as.data.frame(chersX)
chersXD[,2] <- paste("chr",chersXD[,2], sep="")
i <- sessionInfo()
write(paste("#Package:", i$otherPkgs$Ringo$Package), file="/home/paula/GenomeCAT7//test_AG060__160708_033816279_hg18_Spots_RINGO.result")
write(paste("#Version:", i$otherPkgs$Ringo$Version), append=TRUE, file="/home/paula/GenomeCAT7//test_AG060__160708_033816279_hg18_Spots_RINGO.result")
write(paste("#Reference:", gsub("\n", "", i$otherPkgs$Ringo$Reference)), append=TRUE, file="/home/paula/GenomeCAT7//test_AG060__160708_033816279_hg18_Spots_RINGO.result")
write("#normalize: nimblegen",append=TRUE, file="/home/paula/GenomeCAT7//test_AG060__160708_033816279_hg18_Spots_RINGO.result")
write(paste("#Distance:", d),append=TRUE, file="/home/paula/GenomeCAT7//test_AG060__160708_033816279_hg18_Spots_RINGO.result")
write(paste("#nofProbes:", nprobes),append=TRUE, file="/home/paula/GenomeCAT7//test_AG060__160708_033816279_hg18_Spots_RINGO.result")
write("#smooth: RunningMedian",append=TRUE, file="/home/paula/GenomeCAT7//test_AG060__160708_033816279_hg18_Spots_RINGO.result")
write(paste("#winhalfsize:", dist),append=TRUE, file="/home/paula/GenomeCAT7//test_AG060__160708_033816279_hg18_Spots_RINGO.result")
write(paste("#min.probes:", nprobes),append=TRUE, file="/home/paula/GenomeCAT7//test_AG060__160708_033816279_hg18_Spots_RINGO.result")
write(paste("#threshold:", y0G),append=TRUE, file="/home/paula/GenomeCAT7//test_AG060__160708_033816279_hg18_Spots_RINGO.result")
write(paste("#minProbesInRow:", nprobes),append=TRUE, file="/home/paula/GenomeCAT7//test_AG060__160708_033816279_hg18_Spots_RINGO.result")
write(paste("#distCutOff:", 4*dist),append=TRUE, file="/home/paula/GenomeCAT7//test_AG060__160708_033816279_hg18_Spots_RINGO.result")
write.table(chersXD, file="/home/paula/GenomeCAT7//test_AG060__160708_033816279_hg18_Spots_RINGO.result", quote = FALSE, append=TRUE, sep="\t", row.names=FALSE, col.names=FALSE)

**import ChIP-seq data as BAM-Files unsorted, with read shift and poisson based cutoff**
library('Rsamtools', logical.return = TRUE)
library('chipseq', logical.return = TRUE)
library('rtracklayer', logical.return = TRUE)

#read bam files

#read only reads with distinct start positions to avoid artefacts, sort files

param <- ScanBamParam(flag=scanBamFlag(isPaired = FALSE,isDuplicate=FALSE),what=c('pos', 'flag'))
d <- readBamGappedAlignments('/home/paula/GenomeCATProWORK/data/test/SRR037628.bam', index='/home/paula/GenomeCATProWORK/data/test/SRR037628.bam', param=param); d
rd <- unlist(grglist(d));

#handle strand-specific reads to cover potential binding sites

# 1 shift reads by the estimated average distance between
#strand specific peaks in order to center peaks around the “real” binding location - reasonable for isolated short #binding sites.

# 2 extend reads by the estimated average length of DNA fragments – reasonable to cover entire regions of #broader binding sites.

#

#here is version 1 implemented, i.e. shift reads

length_d <-length(d)/1000000;length_d
lss <- estimate.mean.fraglen(rd, method='SISSR'); lss
lscov <- estimate.mean.fraglen(rd, method='coverage'); lscov
lmax <- max(c( median(lss), median(lscov))); lmax
rd <- c(shift(rd[strand(rd) == '+'],lmax/2), shift(rd[strand(rd) == '-'],lmax/2));start(rd[start(rd) < 1]) <- 1

#

#get genomewide coverage
rd_cov <- coverage(rd)
options('scipen'=100)

#

#calculate cutoff value to differentiate peaks from noise.

# 1 extract peaks using a quantile based cutoff. Set cutoff to the value of the 999th permille (999/1000 quantile)

#2 extract peaks using cutoff based on Poisson distribution. calculate cutoff value for given FDR (p = 0.001)

# here is the poisson based peak detection implemented
p_value <- 0.001; cut <- peakCutoff(rd_cov, fdr = p_value); cut

#get peaks with the cutoff value as lower bound
rd_peaks <- slice(rd_cov, lower = cut)

#

#sum up peak coverage, get RPKM
rd_peaks_sum <- peakSummary(rd_peaks)
values(rd_peaks_sum) <- DataFrame(score=((1000*rd_peaks_sum$sum/width(rd_peaks_sum))/length_d))

#

#write out peaks as bedgraph file
write(paste('#shift:', lmax) , append=TRUE, file="/home/paula/GenomeCAT7//SRR03762_peak_NGS_BAM.txt")
write('#peak poisson', append=TRUE, file="/home/paula/GenomeCAT7//SRR03762_peak_NGS_BAM.txt")
write(paste('#p-value:',p_value, '
#cutoff:',cut), append=TRUE, file="/home/paula/GenomeCAT7//SRR03762_peak_NGS_BAM.txt")
export(rd_peaks_sum, format = "bedGraph",append=TRUE, "/home/paula/GenomeCAT7//SRR03762_peak_NGS_BAM.txt")

#

#sum up genomewide coverage per user defined bin-size
rd_bin <- vector('list')
for(i in c(1:length(rd_cov))){
v <- as.vector(rd_cov[[i]])
v <-c(v, rep(0,1000-(length(v)%%1000)))
rd_bin[[names(rd_cov[i])]] <- rowMeans(matrix(v, ncol=1000, byrow = TRUE))*1000/length_d
}

#write binned data as wig file
write('track type=wiggle_0 ', file='/home/paula/GenomeCAT7//SRR03762_bin_NGS_BAM.txt')
write(paste('#shift:', lmax,'
#reads [M]:',length_d) , file="/home/paula/GenomeCAT7//SRR03762_bin_NGS_BAM.txt")
for(i in c(1:length(rd_bin))){
write(paste('fixedStep chrom=', names(rd_bin[i]), ' start=1 step=',1000,' span=',1000, sep=''),append=TRUE, file="/home/paula/GenomeCAT7//SRR03762_bin_NGS_BAM.txt")
write(rd_bin[[i]],sep='\n', append=TRUE, file="/home/paula/GenomeCAT7//SRR03762_bin_NGS_BAM.txt")
}
i**mport ChIP-seq data as BAM-Files with control, calculate differential peaks.**

library('Rsamtools', logical.return = TRUE)
library('chipseq', logical.return = TRUE)
library('rtracklayer', logical.return = TRUE)

#read only reads with distinct start positions to avoid artefacts, sort files

#read experiment data
param <- ScanBamParam(flag=scanBamFlag(isPaired = FALSE,isDuplicate=FALSE),what=c('pos', 'flag'))
d <- readBamGappedAlignments('/home/paula/GenomeCATProWORK/data/test/SRR037628.sorted.bam', index='/home/paula/GenomeCATProWORK/data/test/SRR037628.sorted.bam', param=param); d
 rd <- unlist(grglist(d));
length_d <-length(d)/1000000;length_d

#read control data
c <- readBamGappedAlignments('/home/paula/GenomeCATProWORK/data/test/SRR520469.sorted.bam', index='/home/paula/GenomeCATProWORK/data/test/SRR520469.sorted.bam', param=param); c
rc <- unlist(grglist(c));
length_c <-length(c)/1000000;length_c

#get genomewide coverage for experiment data and control
rd_cov <- coverage(rd)
rc_cov <- coverage(rc)
options('scipen'=100)

#calculate cutoff value for experiment data to differentiate peaks from noise .

# 1 extract peaks using a quantile based cutoff. Set cutoff to the value of the 999th permille (999/1000 quantile)

#2 extract peaks using cutoff based on Poisson distribution. calculate cutoff value for given FDR (p = 0.001)

# here is the poisson based peak detection implemented

p_value <- 0.001; cut <- peakCutoff(rd_cov, fdr = p_value); cut

#get peaks with the cutoff value as lower bound
rd_peaks <- slice(rd_cov, lower = cut)

#sum up peak coverage, get RPKM
rd_peaks_sum <- peakSummary(rd_peaks)
values(rd_peaks_sum) <- DataFrame(score=((1000*rd_peaks_sum$sum/width(rd_peaks_sum))/length_d))

#export experiment data based peaks into bedgraph file
write('#peak poisson', append=TRUE, file="/home/paula/GenomeCAT7//SRR037628.sorte_peak_NGS_BAM.txt")
write(paste('#p-value:',p_value, '
#cutoff:',cut), append=TRUE, file="/home/paula/GenomeCAT7//SRR037628.sorte_peak_NGS_BAM.txt")
export(rd_peaks_sum, format = "bedGraph",append=TRUE, "/home/paula/GenomeCAT7//SRR037628.sorte_peak_NGS_BAM.txt")

#calculate cutoff value for control data to differentiate peaks from noise .

p_value_control <- 0.001; cut_control <- peakCutoff(rc_cov, fdr = p_value); cut_control

#get peaks with the cutoff value as lower bound

rc_peaks <- slice(rc_cov, lower = cut_control)

#sum up peak coverage, get RPKM
rc_peaks_sum <- peakSummary(rc_peaks)
values(rc_peaks_sum) <- DataFrame(score=((1000*rc_peaks_sum$sum/width(rc_peaks_sum))/length_c))

#export experiment data based peaks into bedgraph file

write('#peakControl', file="/home/paula/GenomeCAT7//SRR037628.sorte_peak_control_NGS_BAM.txt")
export(rc_peaks_sum,format = "bedGraph",append=TRUE,"/home/paula/GenomeCAT7//SRR037628.sorte_peak_control_NGS_BAM.txt")

#get difference between experiment data peaks and control data peaks as normalized log2 ratios

#see manual R Module chipseq
peak_d_c <- diffPeakSummary(rd_peaks, rc_peaks)
peaks_diff <- within(peak_d_c, {
diffs <- log2(sums1) - log2(sums2)
resids <- (diffs - median(diffs)) / mad(diffs)
up <- resids > 2
down <- resids < -2
change <- ifelse(up, "up", ifelse(down, "down", "flat"))
})
peaks_diff_exp <- peaks_diff
values(peaks_diff_exp) <-DataFrame(score = (peaks_diff_exp$resids));

#export experiment data versus control data peaks as bedgraph
write('#peakVsControl', file="/home/paula/GenomeCAT7//SRR037628.sorte_peak_vs_control_NGS_BAM.txt")
export(peaks_diff_exp, format = "bedGraph", append=TRUE, "/home/paula/GenomeCAT7//SRR037628.sorte_peak_vs_control_NGS_BAM.txt")
rd_bin <- vector('list')
for(i in c(1:length(rd_cov))){
 v <- as.vector(rd_cov[[i]])
v <-c(v, rep(0,1000-(length(v)%%1000)))
rd_bin[[names(rd_cov[i])]] <- rowMeans(matrix(v, ncol=1000, byrow = TRUE))*1000/length_d
}
